# The veterinary referral process in the DACH region: survey-based insights into obstacles and motivations

**DOI:** 10.3389/fvets.2026.1748365

**Published:** 2026-10-01

**Authors:** C. Gast, M. J. Hassenstein, C. Kleinsorgen, C. Busse, H. A. Volk

**Affiliations:** 1 Department of Small Animal Medicine and Surgery, University of Veterinary Medicine Hannover, Hannover, Germany; 2 Institute of Animal Genomics, University of Veterinary Medicine Hannover, Hannover, Germany; 3 Unit for Research Data Management, University of Veterinary Medicine Hannover, Hannover, Germany; 4 Centre for Teaching, E-Learning Services, University of Veterinary Medicine Hannover, Hannover, Germany

**Keywords:** communication, general practitioner, information, referral, specialist, veterinary medicine

## Abstract

**Background:**

As demand for specialised veterinary care increases, referrals between first opinion practices and specialty clinics are becoming an increasingly important part of the chain of care. However, care pathways between these two settings can involve multiple transitions back and forth, each representing a potential opportunity for error or information loss. Issues such as communication failures, lack of information transfer, and delayed discharge letters back to the first opinion practice may occur, potentially compromising continuity and quality of care. Characterising veterinary surgeons’ satisfaction with this process is therefore an important first step towards identifying where these risks arise and how they might be addressed.

**Objectives:**

This study aimed to evaluate the status quo of the referral process and veterinary surgeons’ satisfaction with it in the German-speaking DACH region (Germany, Austria, Switzerland), in order to identify communication and information gaps along the referral pathway and inform recommendations for improving collaboration between first opinion practices and specialty clinics.

**Methods:**

An online questionnaire was distributed among small animal vets in Germany, Austria and Switzerland. Data from 657 general practitioners (GPs) and specialists (SPs) were analysed.

**Results:**

The majority of veterinary surgeons were generally satisfied with collaboration within the referral process; however, GPs rated cooperation more favourably than SPs. A clear lack of information within referral letters was identified, including medical history, medication details, clients’ contact details, and specific clinical questions. Participants considered good communication, professional skills, and timely discharge referral letters to be the three most important aspects of collaboration. According to GPs, discharge letters were received in 90% of cases, while according to SPs, discharge letters were sent in 80% of cases. The most common problems identified were delayed discharge referral letters (56%), communication failures (55.7%), and missing information (49.5%).

**Conclusion:**

Although participants expressed overall satisfaction with collaboration in the chain of care, problems arise at several points along the referral pathway in the DACH region. We recommend the development of unified referral guidelines within good clinical practice schemes, ideally regulated by relevant authorities, specifically addressing information exchange within referral letters.

## 1 Introduction

The demand for high-level medical care in veterinary medicine is increasing, underscoring the need for comprehensive and effective care (1). One key factor contributing to this trend is the rapid growth in the number of companion animals over the past 20 years. Furthermore, the human-animal relationship has undergone significant changes, with dogs and cats increasingly being viewed as family members or even substitutes for children or partners in some households (2). As a result, pet owners have come to expect a higher level of care from veterinarians, mirroring the standards found in human medical care. This shift has led to an increase in the time required per patient and owner, placing greater demands on veterinary services (3). Early referral has also been shown to carry significant benefits from the client perspective. Regardless of financial background, the majority of pet owners perceived referral as good value for the costs incurred and reported positive outcomes in 70% of cases. Moreover, clients whose general practitioners (GPs) demonstrated a willingness to engage in collaborative care showed a markedly improved perception of their GP, with positive evaluations being up to six times more likely following referral (4).

A strong veterinary system and a good interdisciplinary collaboration between veterinarians are crucial for high-quality veterinary healthcare. In human and veterinary medicine, GPs typically provide general care for common health issues, minor surgeries, and basic services such as vaccinations and check-ups. They also coordinate referrals to specialised veterinarians when necessary. Specialists (SPs), on the other hand, provide advanced, specialised care for specific health issues, including major surgeries, advanced internal medicine, and dental medicine. In certain cases, GPs may refer patients within the chain of care to SPs if they are unsure of how to proceed or if the animal requires specialised treatment. The SP will then provide further diagnostics and/or surgery before discharging the patient back to the first opinion practice for further treatment (5). Referrals from first opinion practices, operated by the GP, to specialised practices and back are an important tool for ensuring that every animal receives the best possible treatment (6). Research has shown that pet owners are more satisfied when referred early through good collaboration between GPs and SPs (4). Therefore, referrals are essential for the collaboration between GPs and SPs, as well as for pets and their caregivers.

Problems with the referral process have been revealed (7–9). One of the primary problems is the inadequate exchange of information between first opinion practices and specialised referral centres, despite the fact that this information may be essential for subsequent care (7, 9). Communication is a key factor within the referral process (10, 11). Failures in communication between GPs and SPs, such as relying solely on spoken information or providing delayed written discharge summaries, can disrupt the continuity of patient care. Communication gaps within the referral process have been associated with disrupted diagnostics, redundant testing, additional visits, and dissatisfaction among pet owners (11). Understanding the referral process itself and identifying potential obstacles can improve the collaboration between GPs and SPs, ultimately resulting in better veterinary care (9, 12). On this basis, it may be possible to create unified referral guidelines, such as the 2025 AAHA referral guidelines, which aim to optimise the process itself and enhance interdisciplinary collaboration between GPs and SPs (12).

The veterinary referral process requires analysis through scientific methods. Several research studies in veterinary and human medicine have been conducted to explore contributing and limiting factors in referral practices in English-speaking countries (13–17). Two studies examined the factors that influence GPs’ choices when selecting an SP for their patients (13, 17). They found that factors such as clinical expertise, good relationships and communication skills were considered important for choosing the right SP. Research on the human medical referral process has also highlighted the following issues: unspecified reasons for referral, lack of information transfer, and unclear expectations of the GP-SP collaboration (14). Problems and solutions for enhancing patient referrals were identified and categorised into clinical decision making, information management, management of patient flows, and quality-of-care monitoring in another examination (15). Deficiencies in clinical decision making can lead to errors in referral necessity, appropriateness, timeliness, and communication. Poor information management is associated with technological communication breakdowns and inadequate data capture, ultimately impacting quality-of-care monitoring.

To date, no studies have been published on veterinary referrals in the German-speaking region (DACH region, Germany (D), Austria (A), Switzerland (CH)). Until now, the perceptions and opinions of German veterinarians have been unclear, particularly regarding obstacles and motivations that may arise, as well as the way the referral process is organised and experienced. As veterinary practice arrangements, processes, training systems, and cultural approaches differ between countries, a direct comparison to referral processes in other countries, especially English-speaking ones, is not possible without further data.

Care for a referred patient can move back and forth several times between the first opinion practice and the specialty clinic—from initial referral, through specialist diagnostics and treatment, to discharge and follow-up care by the GP—and each of these transitions represents a potential point at which information can be lost or miscommunicated (Figure 1). Characterising how satisfied veterinary professionals are with this process, and where specifically it breaks down, is therefore an important step towards identifying targeted improvements. The aim of this study was to survey the current practice of the veterinary referral process in German-speaking countries (DACH region). Specifically, it examines how veterinary professionals collaborate across the referral pathway and how satisfied they are with the process. Particular attention is paid to the implementation of discharge referrals, as this aspect of the process may harbour potential for professional tension between GPs and SPs. In addition, this study investigates whether critical or negative attitudes toward the referral process exist among veterinary professionals. A central hypothesis is that inadequate communication between referring and receiving veterinarians constitutes one of the primary weaknesses of the referral process. Based on the findings, this study aims to serve as basis for recommendations for standardised referral guidelines to support collaboration between GPs and SPs.

**Figure 1:**
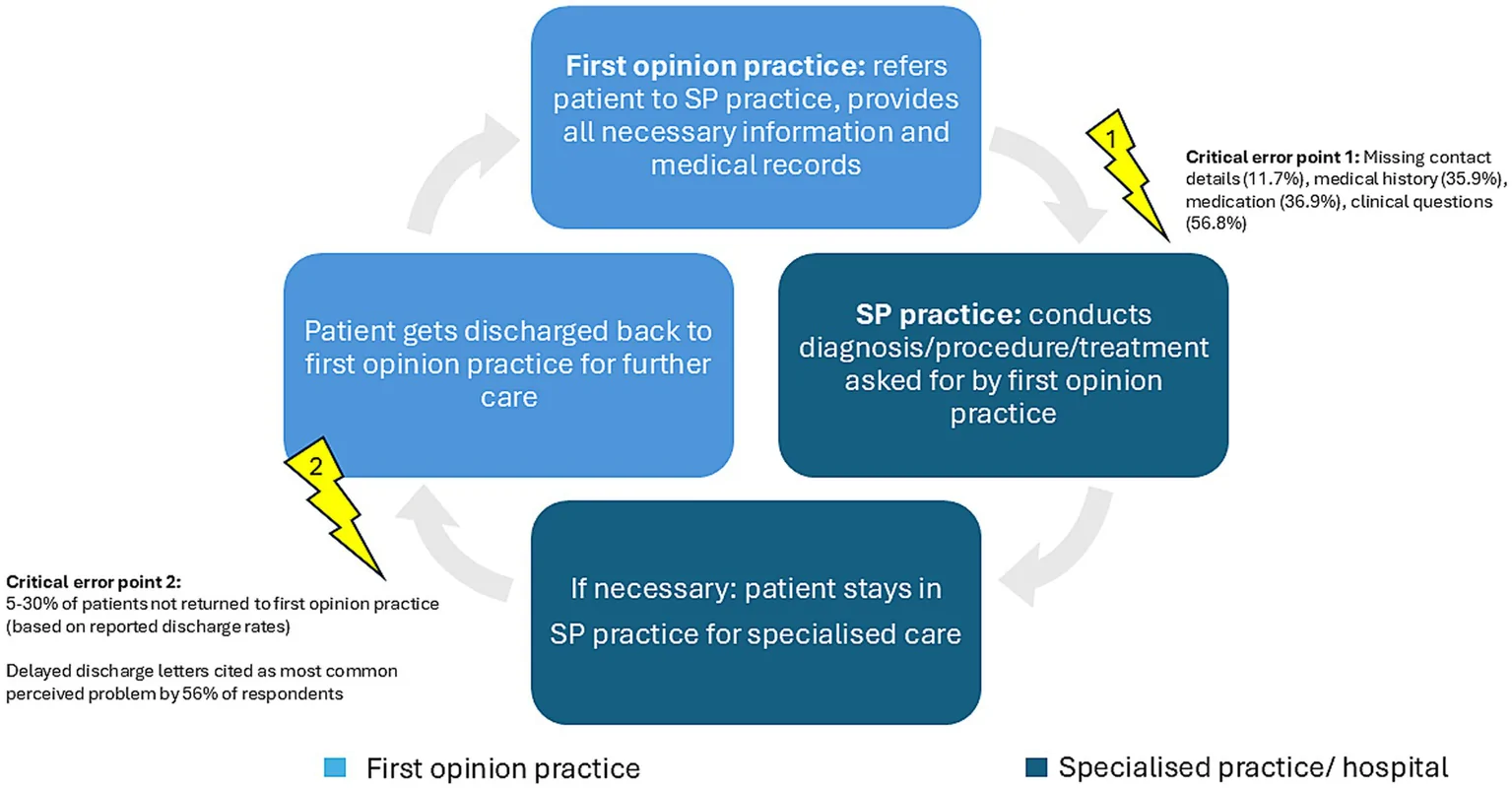
Conceptual overview of the veterinary referral cycle and its critical error points. Care for a referred patient moves cyclically between the first opinion practice and the specialist (SP) practice: the general practitioner refers the patient together with the relevant clinical information and medical records; the SP practice performs the diagnostics, procedure or treatment requested; and the patient either remains in specialised care where necessary or is discharged back to the first opinion practice for further care. Two transitions carry the greatest risk of information loss (lightning symbols). At the point of referral, specialists reported not receiving owner contact details in 11.7% of referrals, medical history in 35.9%, medication details in 36.9% and the specific clinical questions to be addressed in 56.8% (lightning symbol 1). At the point of discharge, 5–30% of referred patients were not returned to the first opinion practice, and delayed discharge letters were the most frequently reported problem overall (56% of respondents) (lightning symbol 2).

## 2 Methods

### 2.1 Study sample

The target population for the survey was defined as veterinarians currently practising in small animal medicine, regardless of age or gender. To ensure that the survey reached the intended audience, inclusion criteria were established so that only small animal veterinarians actively practising in the DACH region (Germany, Austria, Switzerland) were eligible to participate. Conversely, exclusion criteria were established to exclude veterinarians who did not meet the target population criteria, including veterinarians specialising in large animal or exotic medicine, retired veterinarians, and veterinarians working in industry or as governmental veterinary officers.

The veterinary profession in the DACH region is characterised by a strong female majority (18), which is expected to be reflected in the composition of the sample.

### 2.2 Questionnaire design and content

A questionnaire was designed to collect data from the target population. The questionnaire was divided into distinct sections, with questions tailored to either GPs or SPs, depending on the participant’s self-identification. This approach allowed for the collection of relevant data specific to each group, facilitating a more nuanced understanding of the referral process in small animal medicine.

The questionnaire was developed based on previous studies within the research field (19–21), with the majority of the content created by the authors. However, we drew inspiration from existing questionnaires to inform our design. A study’s questionnaire prompted us to ask GPs and SPs about their satisfaction with professional collaboration and whether they would recommend the GP/SP to a colleague (19). Unlike the original study, we chose to formulate the questions more openly to accommodate the exploratory nature of our survey. Another examination influenced our decision to ask for the most common problems within the referral process (20). We adapted their question about the timeliness, complexity, and appropriateness of referrals to explore potential issues within the DACH referral process. This redesign allowed us to identify a rough direction for potential problems, providing a foundation for further research. Following the example of Rosemann et al. we included questions about the information contained in referral letters or forms, such as the patient’s medical history, prescriptions and specific requests to the SP (21). The remaining questions were entirely constructed by us.

The questionnaire was divided into three sections. The first section gathered basic, non-sensitive demographic information such as age and gender. Participants were given the option to decide whether to release this information or not. The second section consisted of four questions that focused on the professional background of the participants, including:

- The type of veterinary practice they currently work in
- The species they treat
- A distinction between GPs and SPs
- The average number of patients seen per week

Sections three and four were tailored according to the participant’s self-identified role as either a GP or an SP, allowing us to collect relevant data specific to each group.

### 2.3 GP questionnaire

To gather data on the referral process, we first categorised GPs into two groups: those who regularly refer patients to specialty practices and those who do not or have never referred patients. The participants who regularly refer patients were asked to complete a series of multiple-choice questions about the referral process, including information transfer, their satisfaction with the collaboration with the referral practice, and whether they would recommend the referral practice to a colleague (on a scale from 1 to 10, using the Net Promoter Score). This allowed us to assess the GPs’ experiences and perceptions of the referral process.

To investigate a commonly discussed issue in the referral process – the discharge of patients back to the first opinion practice – we asked GPs how many of their patients they typically receive back from the specialist practice. This question aimed to provide insight into the frequency and volume of discharge referral letters. Additionally, we included an open-ended question to allow GPs to share their thoughts on important aspects of the referral process, providing a more nuanced understanding of their experiences and challenges.

### 2.4 Specialist questionnaire

SPs received a series of questions that were similar to those asked of GPs, but with some modifications to accommodate their unique perspective. The specialists’ section began with questions regarding referral frequency and the typical content of referral documentation they receive. This allowed us to understand the volume and nature of referrals they receive. Participants were then asked to rank the importance of various pieces of information that are typically included in referral documentation, such as the owner’s name and contact information, medical history, current medications, and specific requests from the referring GP. To facilitate comparison with the GPs’ responses, we grouped specialists’ Net Promoter Score answers from important to essential as “important” in our diagram (Figure 2), rather unimportant to rather important as “moderate,” and extremely unimportant to unimportant as “unimportant.”

**Figure 2:**
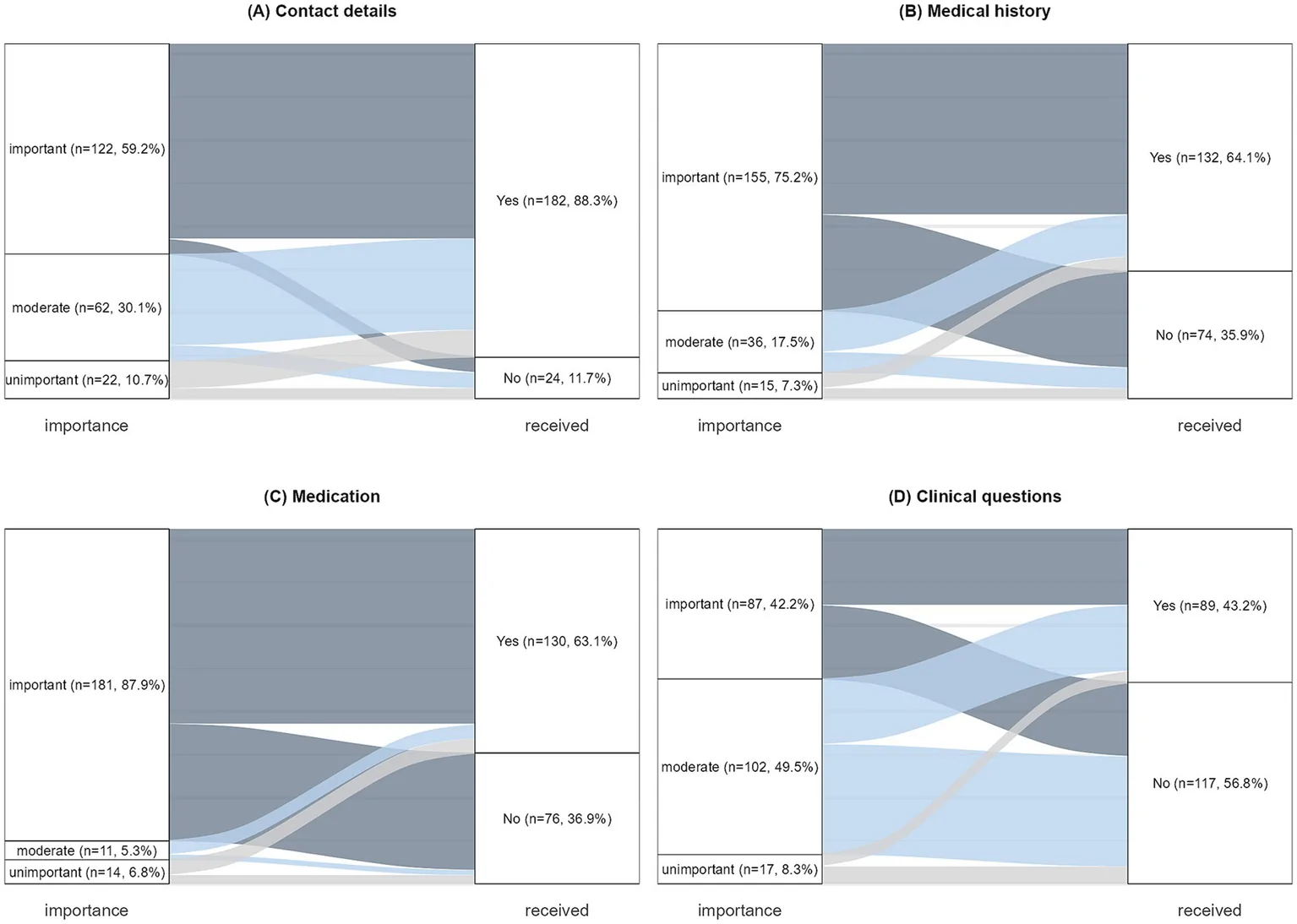
Information in referral forms (Multiple Choice) & Importance of information within referrals (Single Choice). Left legend: Scores from 1 (Extremely unimportant) to 10 (Extremely important). Labels 8–10 “important” = Net promoters, Labels 4–7 “moderate,” Labels 1–3 “unimportant.” Right legend: Received (“Yes) or not received (“No). Dark blue wave: Group of participants that consider this information as “important” = Net promoters. Light blue wave: Group of participants that consider this information as “moderate.” Grey wave: Group of participants that consider this information as “unimportant”.

As with GPs, specialists were asked to evaluate their working relationship with referring practitioners. Some questions focused on potential improvements in the GP-specialist relationship, with participants asked to select the three most important aspects of collaboration from the same list provided to GPs.

From this point onwards, the questions for SPs diverged from those for GPs. They were asked to rate how likely they would be to recommend their referring GP to colleagues, on a scale from 1 to 10, using the Net Promoter Score. This allowed us to assess the specialists’ perceptions of the referring GP’s quality and reliability. To assess the frequency of discharge referral letters back to the first opinion practice from the SP’s perspective, participants estimated the percentage of referred patients who were subsequently returned to the GP.

Finally, both target groups were asked about the most common problems within the referral process, with options including delayed discharge referral letters, insufficient communication, missing information, and dissatisfaction among the patient’s owners. This allowed us to identify potential areas for improvement in the referral process and to compare the perspectives of GPs and specialists.

### 2.5 Study tool

To distribute the questionnaire, we utilised the online platform LimeSurvey® (LimeSurvey Community Edition Version 6.13.2 + 250,506, LimeSurvey GmbH, Hamburg, Germany), which is accessible via all common internet-enabled devices. Prior to the main data collection, the questionnaire was pilot tested with five participants, who provided feedback on technical functionality, item clarity, and content comprehensiveness. Minor revisions were made accordingly before the survey was distributed to the full sample. The survey was launched in February 2025 and remained open until the end of April 2025. Participants were provided with a unique URL that granted them access to the online survey, allowing them to complete it immediately or later. Participation in the survey was entirely voluntary. Completing the questionnaire required approximately 10–15 min.

To ensure broad dissemination, the survey was promoted through multiple veterinary channels. The survey was distributed via the German veterinary chambers’ newsletter, as well as through leading companies and individuals in the veterinary sector. Additionally, it was advertised through the social media accounts of the University of Veterinary Medicine Hannover (TiHo) and shared by various veterinary-related accounts. Furthermore, the questionnaire was shared in relevant social media groups for veterinarians. To reach a wider audience, key veterinary organisations and practices were contacted and asked to circulate the survey within their network and among their veterinary staff. Finally, the survey was published in the “Deutsches Tierärzteblatt,” the official journal received monthly by all licensed veterinarians in Germany.

### 2.6 Analysis

Once the survey period ended, the data were exported from LimeSurvey as a spreadsheet (MS Excel) to be further checked in terms of completeness and plausibility. We excluded all participants who only answered the demographic questions in the first section, as these responses did not provide any relevant information for our study.

Our analysis focused on the questions that assessed the collaboration between GPs and SPs, including the likelihood of recommendation to a colleague. We also examined the questions related to information transfer and the importance of information, particularly the types of information included in referral forms/letters and how SPs rate the importance of certain information. In addition, we evaluated data on the number of discharge referral letters back to the first opinion practice from both the SPs and GPs perspectives. To facilitate comparison, we grouped similar questions from GPs and SPs into figures or combined them into a single figure. To summarise the Net Promoter Score questions about the information received by GPs and the importance of certain information, we categorised the responses as follows:

- “Important”: Labels 8–10
- “Moderate”: Labels 4–7
- “Unimportant”: Labels 1–3

This categorisation allowed us to create a clear and comparable figure (Figure 2) that summarised the collected data. All data preparation and analysis were conducted using R version 4.4.0 (22), and visualisations were built using ggplot2 package (23).

### 2.7 Ethical considerations

The study protocol was reviewed internally by the institutional review board and responsible data protection officer at the University of Veterinary Medicine Hannover, who approved the study. Data collection and analysis complied with relevant data protection regulations. This study did not receive any external funding.

## 3 Results

### 3.1 Population

A total of 822 veterinarians participated in our survey. However, due to incomplete responses or early termination of the questionnaire, we excluded 165 participants. This left us with a sample size of 657 small animal veterinarians, comprising 451 GPs and 206 SPs who work in a referral practice or hospital (Table 1). The age distribution of our participants was as follows: 33 participants (5.0%) fell into the 20–29 year age group, comprising 15 GPs (3.3%) and 18 SPs (8.7%). In contrast, only 1.1% (5/451) of the GPs stated they were between 70 and 79 years old, while none of the SPs fell within this age group. Additionally, 1.1% (7/657) of respondents preferred not to answer the age question.

**Table 1:** Population characteristics.

	Overall *N* = 657	General practitioners *n* = 451	Specialists *n* = 206
Age group			
20–29 years	33 (5.0%)	15 (3.3%)	18 (8.7%)
30–39 years	147 (22%)	79 (18%)	68 (33%)
40–49 years	161 (25%)	111 (25%)	50 (24%)
50–59 years	218 (33%)	175 (39%)	43 (21%)
60–69 years	86 (13%)	60 (13%)	26 (13%)
70–79 years	5 (0.8%)	5 (1.1%)	0 (0%)
Not specified	7 (1.1%)	6 (1.3%)	1 (0.5%)
Gender			
Male	99 (15%)	60 (13%)	39 (19%)
Female	548 (83%)	383 (85%)	165 (80%)
Not specified	10 (1.5%)	8 (1.8%)	2 (1.0%)
Treated animal species (multiple answers possible)			
Dogs and cats	657 (100%)	451 (100%)	206 (100%)
Rodents	519 (79%)	386 (86%)	133 (65%)
Equines	68 (10%)	56 (12%)	12 (5.8%)
Bovines	53 (8.1%)	50 (11%)	3 (1.5%)
Swines	21 (3.2%)	20 (4.4%)	1 (0.5%)
Birds	142 (22%)	110 (24%)	32 (16%)
Exotics	78 (12%)	52 (12%)	26 (13%)
Patient’s per week per vet, Median (Q1, Q3), Mean	Median: 60 [50, 100]; Mean: 77 (60)	Median: 75 [50, 100]; Mean: 85 (58)	Median: 50 [35, 60]; Mean: 59 (60)

Our study sample consisted of 548 females (83.4%) and 99 males (15.1%), 10 participants (1.5%) preferred not to state their gender. The participants treated an average of 77 animals per week, with a mean of 59 pets in specialty practices and 85 animals in general practices.

### 3.2 Satisfaction with referral process/ interdisciplinary cooperation

The majority of GPs rated the cooperation with the referral practices as “Very good” (25.6%) or “Good” (25.4%), compared with 12.6% (“Very good”) and 27.7% (“Good”) of SPs. In total, 20.4% (90 out of 441) of the GPs rated the cooperation as “acceptable” or “fair,” whereas 37.4% (77 out of 206) of SPs evaluated the cooperation as “acceptable” or “fair” (Figure 3).

**Figure 3:**
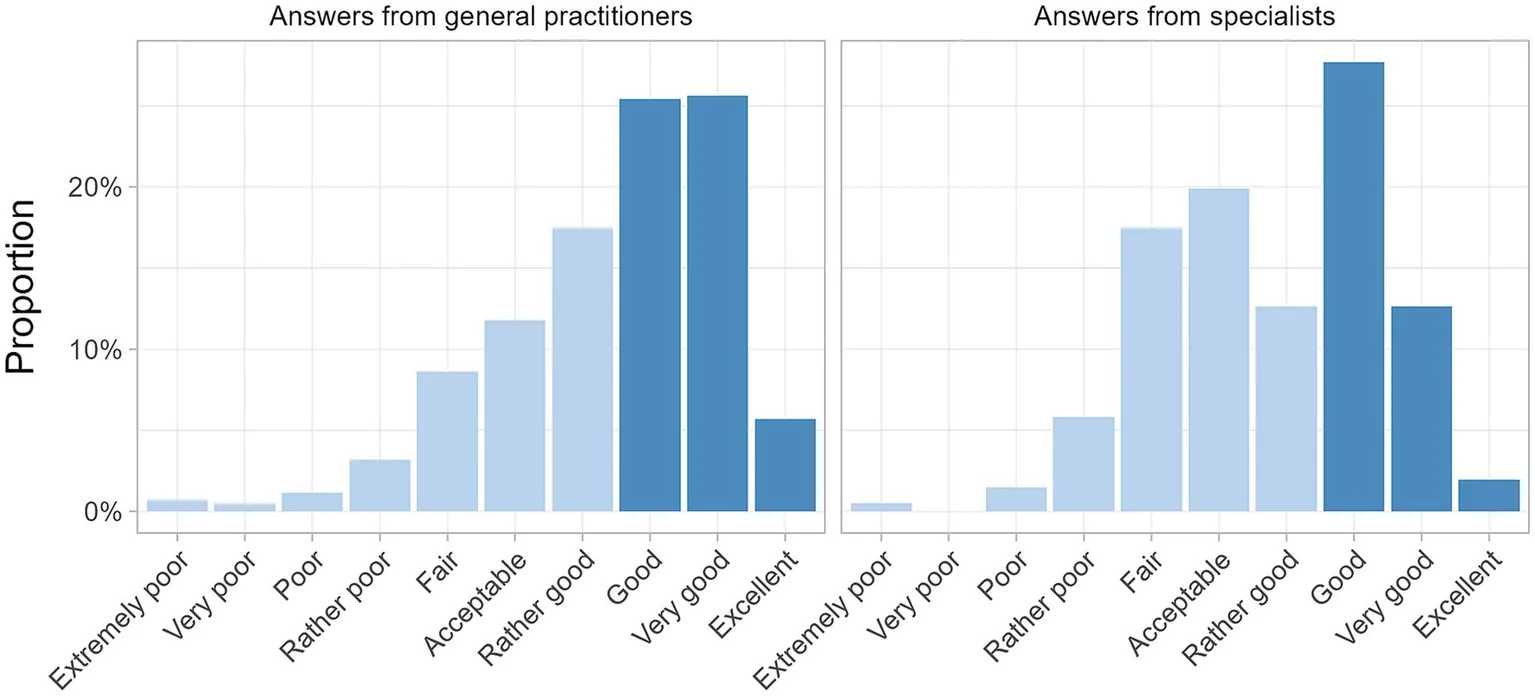
Rating of the perceived level of cooperation with the referral and first opinion practices (Single Choice; 1 (Extremely poor) to 10 (Excellent); Darker blue = Net Promoters). General practitioners’ net promotor score was 5.4 and the specialist one was only −30.7.

The majority of SPs (71.3%) indicated they would recommend the collaborating GP, with ratings ranging from “moderately likely” to “extremely likely”; however, the largest share selected “moderately likely.” The diagram (Figure 4) shows that SPs’ recommendations of GPs are evenly distributed. In contrast, GPs’ recommendations of SPs’ cluster on the positive side: 36.8% selected “very likely,” 24.4% “extremely likely,” and 20.6% “likely.”

**Figure 4:**
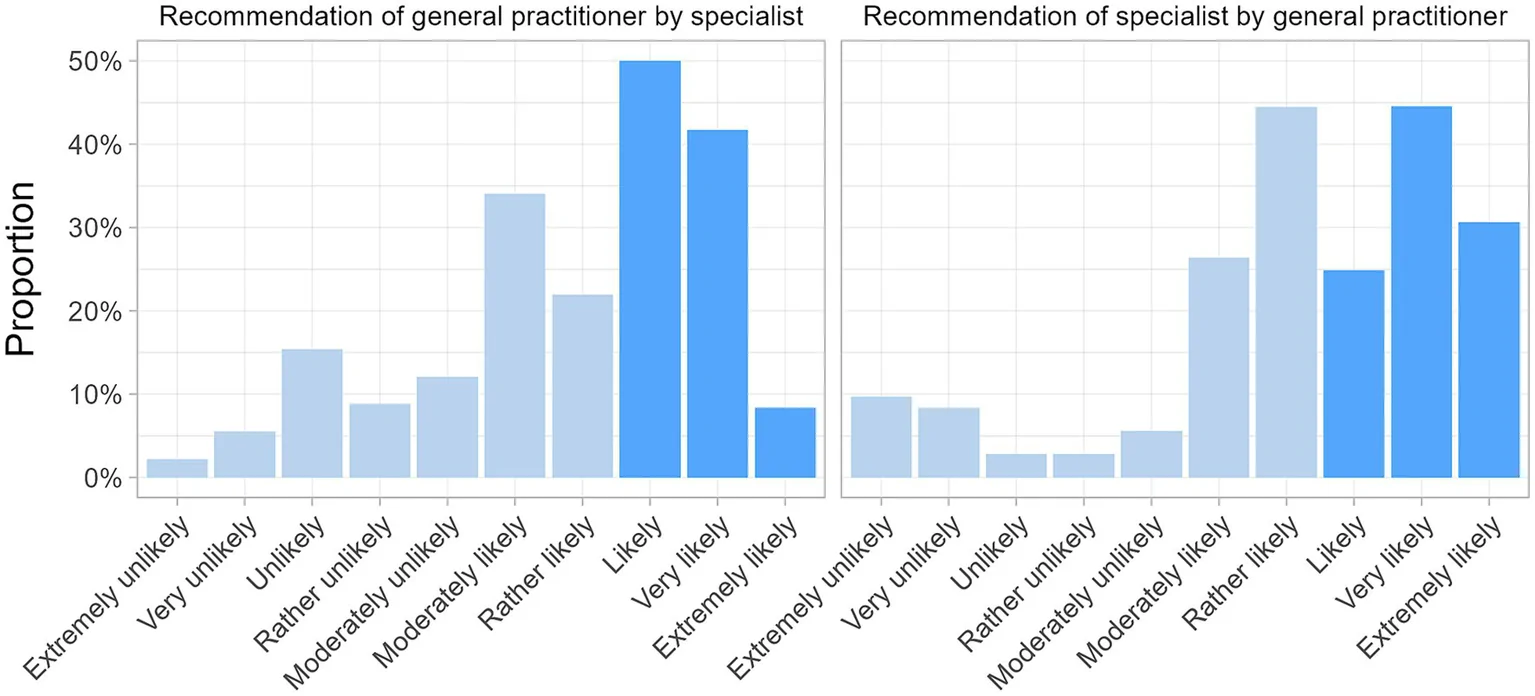
Recommendation of specialist/ general practitioner (Single Choice), Net Promoterscore from 1 (Extremely unlikely) to 10 (Extremely likely), Darker blue = Net Promoters. General practitioners’ net promotor score was 52.6 and the specialist one was only −33.8.

### 3.3 Information transfer

Accuracy of data regarding information transfer during referrals showed notable differences. Owner contact details were considered important by 59.2% and moderately important by 30.1% of SPs. Overall, 88.3% of SPs reported that they routinely receive owner contact details in referral letters. Among these, 62 SPs (30.1%) considered this information “moderately important,” while 10.7% of SPs rated it as “unimportant.” By contrast, 97.5% of GPs stated that a client’s contact details are usually included in their referral forms.

Medical history was considered important by most SPs, with a median rating of 9 (very important) on a scale of 1–10. However, 64.1% of specialists reported receiving medical history information from referring veterinarians. Notably, 36.1% of specialists who wished to receive information about their patient’s medical history did not receive it. Breaking down the responses further, 17.5% of respondents considered medical history “moderate” in importance, and 66.7% of them received information about medical history. In contrast, 15 SPs rated medical history as “unimportant,” but still received it in 60% of cases. 97.1% of GPs typically include medical history in their referral forms.

Information about previous medication is contained in 95.5% of GPs’ referrals but received by only 63.1% of SPs. Of the 206 SPs asked, 181 (87.9%) considered medication crucial information in referrals but only received it in 62.4% of cases. Fifty percent of responses were at least a rating of 9 (= very important). Only 6.8% of SPs considered medication as unimportant and yet received it in 64.3% of cases.

Specific clinical questions of the GP regarding the patient that should be addressed by the SP within the referral were included by 77.5% of the GPs but actually received by 43.2% of SPs. This discrepancy is highlighted by the fact that 42.2% of SPs (87 out of 206) wished to receive this information, with a median rating of 7 (= rather important) (Figure 2). Furthermore, 62.7% of respondents who classified specific clinical questions as “moderate” in importance did not obtain this information in referral letters. Of the 17 participants (8.3%) who did not need to know about specific clinical questions, 41.2% received it and 58.8% did not.

### 3.4 General aspects of the referral process

There is a clear consensus that three key aspects are crucial for effective collaboration between GPs and SPs (Table 2). Overall, good communication was rated the most important aspect (13.2%), followed by professional skills (13%) and timely discharge referral letters back to the first opinion practice (11.2%). This ranking was consistent among SPs, who rated communication highest (11.6%), followed by professional skills (10.2%) and discharge referral letters (9.6%). Among GPs, however, professional skills was rated marginally higher than communication (11.6% vs. 11.2%), while discharge referral letters was again ranked third (9.6%). GPs were asked, in the context of an open-ended question, what else was important to them within the referral process. Here too, within the answers, the term “communication” was counted most frequently (see Supplementary Figure S1).

**Table 2:** Three most important aspects within the collaboration in care (multiple choice).

Aspects	Total	General practitioners	Specialists
Appropriate treatment	262 (6.7%)	154 (4.9%)	108 (7.5%)
Good communication	517 (13.2%)	350 (11.2%)	167 (11.6%)
Other	–	9 (0.3%)	–
Professional skills	512 (13%)	365 (11.6%)	147 (10.2%)
Proximity	42 (1.1%)	36 (1.1%)	6 (0.4%)
Timely discharge referral letters	441 (11.2%)	302 (9.6%)	139 (9.6%)
Transparent reporting	228 (5.8%)	112 (3.6%)	116 (8%)
Trust	–		109 (7.6%)

Furthermore, both target groups were asked how many patients are discharged back to the general practice. The data show a match between the two groups, with an interval of 70 to 95% each. However, the median differs slightly, with SPs stating that 80% of patients are discharged back to the first opinion practice and GPs indicating that 90% of referred patients are discharged back to them (Figure 5).

**Figure 5:**
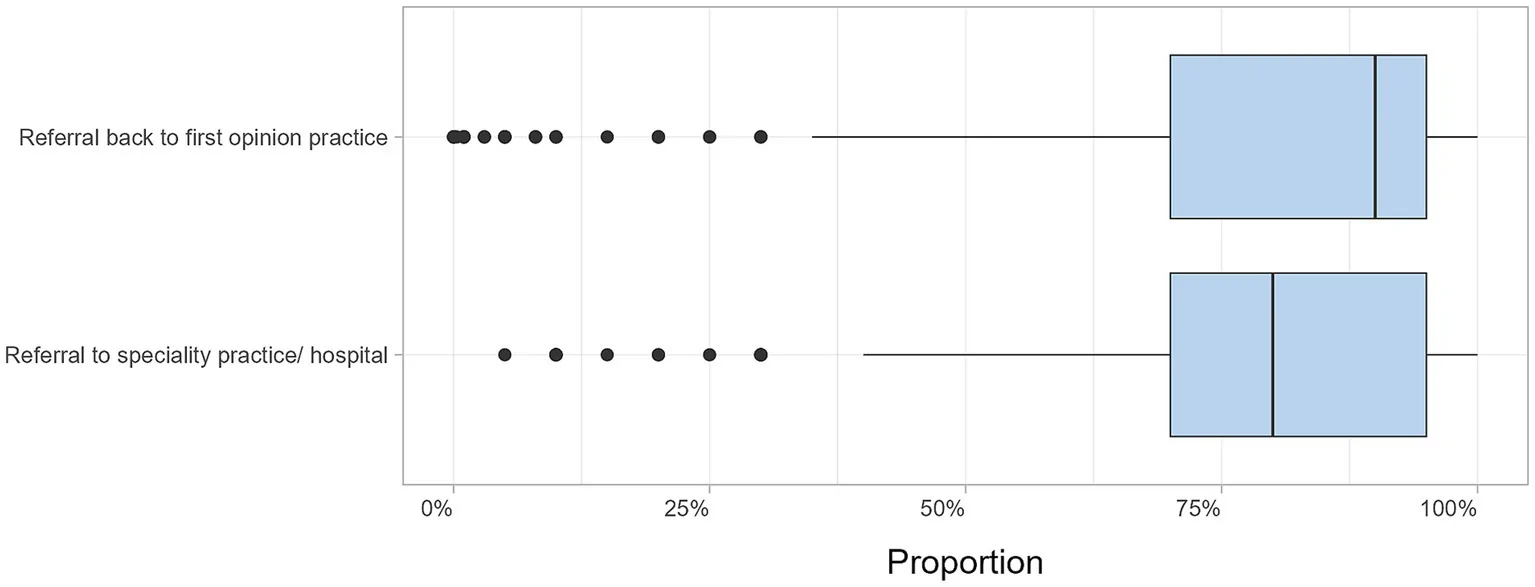
Number of discharged patients received by the GP & Number of patients discharged to the GP by specialist (Numeric input). Median referral to speciality practice/hospital 80%, median referral back to first opinion practice 90%.

More than half of the respondents (56%, *n* = 311) believe that delayed discharge referral letters are the most common problem within the referral process, closely followed by insufficient communication (55.7%, 309 participants). For 49.5% (275) of the participating veterinary surgeons, missing information is one of the most frequent issues (Table 3). Lastly, 34.1% (*n* = 189) stated that discontent of patient owners belongs to the most common problems.

**Table 3:** Most common problems within the referral process (multiple choice).

Problem	Count (n)	Percent (%)
Delayed discharge letters	311	56
Insufficient communication	309	55.7
Missing information	275	49.5
Dissatisfaction of patient owners	189	34.1
Other	70	12.6

## 4 Discussion

The provision of high-quality medical care for pets relies heavily on effective collaboration along the chain of care and a well-functioning referral system. However, identifying areas for improvement is crucial to optimising workflow and ensuring seamless care transitions. This study, based on a survey of veterinary surgeons in the German-speaking DACH region, reveals significant gaps in information transfer during referrals, with essential information frequently absent. SPs reported receiving owner contact details in 88.3% of referrals, medical history in 64.1%, information about current medication in 63.1%, and the specific clinical questions to be addressed in only 43.2% of cases. These findings underscore the need for improved communication and data exchange protocols to enhance the quality of care for pets.

Medical referrals between GPs and SPs are central to ensuring a high quality of veterinary medicine (12). GPs refer their clients to SPs to provide the best possible care for the patient and to ensure a positive client experience. Conversely, SPs rely on referrals to focus on their area of expertise and to allocate their resources effectively to referred patients (14). The smooth functioning of the referral process relies on good communication, cooperation, and information transfer between the two parties. However, the referral system in veterinary medicine is not always seamless, and challenges can arise when GPs and SPs do not work together effectively (7).

Our survey revealed that many referral letters lack essential information that SPs need to provide optimal care for patients. Specifically, we found that owners’ contact details, information about current medication, and medical history were often missing from referral letters. This finding is consistent with several studies in human medicine, which have also highlighted the importance of complete and accurate information in referral letters (14). A comparison of our findings with those of a previous study highlights the scope of the problem (24). In the previous study, a patient’s medical history was included in 85% of all referral letters, whereas in our study, it was only included in 64.1% of cases. Similarly, while current medication was part of the referral letter in 82% of cases in the previous study, it was only covered in 63.1% of patients in our study.

Further research revealed that SPs did not receive the information they needed 23% of the time (9). This is a concerning finding, as it highlights the potential for errors and adverse outcomes in veterinary medicine. Interestingly, a comparison of our findings with those of human medicine suggests that the completeness of information in referral letters is still better in human medicine than in our study. One possible explanation for this difference might be the higher level of responsibility and potential consequences for medical staff in human medicine. In human medicine, medical staff may face more severe consequences, including lawsuits and loss of licensure, if they fail to provide adequate care due to a lack of information in referral letters. In contrast, veterinary medicine may not have the same level of accountability and consequences for medical staff, which could contribute to a lack of attention to this critical issue.

Our study identified communication, timely discharge letters back to the first opinion practice, and professionalism as the three most important factors within the collaboration between GPs and SPs. This finding is consistent with previous research, which has also highlighted the importance of these factors in ensuring effective collaboration between GPs and SPs. One study found that “Effective and timely communication was a strong contributor to how participants viewed the referral experience […]” (25). That study explored the expectations of equine GPs towards equine SPs through qualitative group interviews. Another study found that communication failure was a significant issue, with 80% of analysed cases experiencing some form of communication breakdown (26). This finding highlights the importance of effective communication in ensuring successful referrals and high-quality patient care.

In human medicine, research has also investigated the factors that contribute to the success of referrals. A study conducted in six family practice residency programmes affiliated with the Northeastern Ohio Universities College of Medicine found that GPs who received information about the referral in a letter or form from the SP were more satisfied with patient care and communication (27). This suggests that clear and timely communication is a critical factor in ensuring successful referrals and high-quality patient care. Additionally, good communication eases the collaboration between GPs and SPs. Therefore, communication training is needed not only among experienced senior veterinarians, but already in university for future veterinarians (12, 28). Another human medicine study showed that 88% of respondents considered the medical skill of the SP to be of major importance when it comes to choosing an SP to refer to (29).

Data transfer within referrals should be digitalised, whether in a newly built web-based computer system or integrated into existing software already used in practices, to ensure that every essential piece of information reaches the specialty practice and can easily be fed back also to the first opinion practice, along the chain of care (30, 31). However, ideally in future, electronic information records should consider a triangle of care, including GPs, SPs, and the pets’ caretakers. A participatory approach and transparency of care will be the ideal future veterinary care concept.

Our study found that according to the SPs, 80% of the cases are discharged back to the first opinion practice and 20% are not. Possible explanations might be lack of time for writing a discharge referral letter or dissatisfaction of the patient owner with the previous first opinion practice. The 2025 AAHA Referral Guidelines recommend that SPs should discharge their referred patients to the original first opinion practice (12). Properly conducted discharge referrals can result in increased satisfaction of the GP within the referral process (32).

The findings of this study indicate that there is currently no common consensus on a standardised referral process within the DACH region. To facilitate collaboration between GPs and SPs and to establish greater consistency, unified guidelines could be developed by the relevant veterinary chambers and stakeholder organisations. The 2025 AAHA Referral Guidelines may serve as a valuable starting point in this regard, although adaptation to the specific professional, cultural, and regulatory context of the DACH region would be warranted before implementation (12). Such guidelines should specifically address the points at which this study identified the greatest risk of information loss—namely at the point of referral (missing contact details, medical history, medication information, and specific clinical questions) and at the point of discharge back to the first opinion practice (Figure 1). By explicitly defining minimum requirements for information transfer at each of these transitions, unified guidelines could directly target the main sources of error identified in this study.

Beyond formal guidelines, chambers and organisations should consider providing more structured support for their members, including standardised referral forms, continuing education courses, and webinars. Particular emphasis should be placed on fostering collaborative care, for instance through joint events that bring together GPs and SPs to exchange ideas and address current challenges. At the academic level, the referral process should be integrated more explicitly into veterinary curricula, whether through dedicated lectures in small animal medicine or as part of the clinical practical year.

From a technological perspective, digital referral tools should be incorporated into practice management systems to streamline the exchange of clinical information, including imaging data such as radiographs, CT, and MRI scans, as well as laboratory results. The implementation of a shared digital patient record, accessible to both the referring and receiving veterinarian, could further reduce information loss and improve continuity of care.

## 5 Limitations

As with any online survey, the recruitment methods employed in this study may not have captured all GPs and SPs in the DACH region, and the resulting sample may therefore not be fully representative of the target population. Voluntary participation poses the risk of self-selection bias, whereby the sample may be primarily composed of individuals with a particular interest in the referral process. Similarly, the digital format of the survey may have deterred less digitally experienced veterinarians, potentially leading to their underrepresentation. As all data were based on self-report, responses may be subject to both recall bias and social desirability bias, whereby participants may have responded in a manner they perceived as professionally favourable rather than accurately reflecting their actual practice. Furthermore, the predominantly female sample (83%) may limit the extent to which the perspectives of male veterinarians are represented. The high proportion of female respondents is, however, consistent with the known demographic composition of the veterinary profession in the DACH region, where women represent the majority of practising veterinarians, and the sample is therefore considered broadly representative of the target population (18). Finally, the findings of this study are specific to the DACH region and may not be directly transferable to other cultural or healthcare contexts, given that referral practices, professional norms, and client expectations may differ substantially across countries.

## 6 Conclusion

Understanding how satisfied veterinary professionals are with the referral process and identifying where along the care-pathway problems arise, is an important step towards improving collaboration between first opinion practices and specialty clinics. Our study revealed that participating veterinarians in the DACH region are generally satisfied with the referral process, but there is room for improvement. Specifically, information transfer is often inaccurate and incomplete, with essential details such as medical history and current medication frequently missing from referral letters or forms. Veterinarians identified communication failures and missing discharge referral letters as key obstacles within the referral process.

To address these issues, unified guidelines for the German-speaking region could provide veterinarians with clear guidance on best practices. They have the potential to meaningfully reduce the barriers and inefficiencies currently observed within the referral process. Future research should focus on gathering more information about the reasons and motivations for referrals, to specify the suggestions mentioned above.

## Data Availability

The original contributions presented in the study are included in the article/Supplementary material, further inquiries can be directed to the corresponding author.
