# Supplementary material for: The veterinary referral process in the DACH region: survey-based insights into obstacles and motivations
Source: Front Vet Sci. 2026 Oct 1;13:1748365. doi: 10.3389/fvets.2026.1748365 (PMC13671021; doi:10.3389/fvets.2026.1748365)
Supplement: Supplementary file 1 [file Data_Sheet_1.pdf]

Dear colleagues,

My name is Corinna Gast. I am a newly graduated veterinarian and am currently working on my dissertation on "Referral behaviour in veterinary medicine in the DACH region" at the University of Veterinary Medicine Hannover under the supervision of Professor Holger Volk.

As part of my doctoral thesis, we would like to examine the processes involved in veterinary referrals, exploring both the perspective of pet veterinarians and that of specialists. This will enable us to derive recommendations for improved cooperation and thus more efficient working methods. To do this, we need your help!

We would be very grateful if you could take 10-15 minutes to complete our survey. By doing so, you will be contributing to the improvement of work processes in the field of veterinary medicine!

For the study to be successful, it is important that you complete the questionnaire as fully as possible. Please note that some questions may appear similar. However, each question answered provides different and valuable information, which is why it is essential to answer each one. Your information will be treated confidentially and evaluated anonymously.

This questionnaire does not use gender-neutral language, but is intended to address all genders equally. Thank you for your understanding and participation!

Corinna Gast (Doctoral student) Professor Holger Volk, Dr. Claudia Busse, Dr. Christin Kleinsorgen

Part A: Question group 1 – Personal details

A1. **Which age group do you belong to?** ***(Single selection, voluntary information)***

- 20-29 years ☐
- 30-39 years ☐
- 40-49 years ☐
- 50-59 years ☐
- 60-69 years ☐
- 70-79 years ☐
- Over 80 years ☐

A2.

Which gender do you most identify with?
(Single selection, voluntary information)

- Female ☐
- Male ☐
- Diverse ☐

Part B: Question group 2 – Information about your practice

B1.

Which animal species do you treat? ***(Multiple selections possible)***

- Small animals (dogs and cats) ☐
- Small rodents (e.g. rabbits, guinea pigs) ☐
- Equines ☐
- Bovines ☐
- Swines ☐
- Birds ☐
- Exotic animals ☐

Other

Other

[ ]

B2.

What best describes your veterinary practice?
(Single selection)

- First opinion veterinary practice ☐
- Specialist veterinary practice ☐
- Referral centre/health centre ☐
- Referral centre (hospital) ☐

B3.

How many patients does a vet see in your practice on average per week?
(Numerical input)

[| | | | | | | | |]

Part C: Question group 3 – Veterinary practice

C1.

Do you refer patients to other practices or hospitals*? (Single selection)*

- Yes ☐
- No ☐

C2.

What are the reasons for not using referrals?
(Multiple selection)

- I am unsure when a referral is medically appropriate. ☐
- I am afraid of losing the patient owner ☐
- I lack established contacts with specialized clinics or practices. ☐
- I am afraid that a referral could damage my relationship with my patients' owners. ☐
- I doubt whether the patient's owners can cover the costs of a referral ☐
- There are no specialised facilities in my area ☐

LimeSurvey

- [ ] Organising and implementing referrals is too complex
- [ ] I would like to carry out as many treatments as possible in my own practice
- [ ] I have the necessary diagnostic and therapeutic equipment in my practice, so a referral is not necessary
- [ ] I have concerns about the quality or usefulness of a referral
- [ ] Other

Other

C3.

What changes could persuade you to consider referrals? ***(Multiple selections)***

- [ ] Clear guidelines or recommendations on when a referral is appropriate
- [ ] Access to a list of trusted referral practices or clinics
- [ ] Better communication and cooperation with specialised institutions
- [ ] Support in organising and processing referrals
- [ ] More transparency about costs and treatment options in referral practices
- [ ] Feedback and reports from specialists after a referral
- [ ] Training courses that address the benefits and procedures of referrals
- [ ] Geographically closer referral facilities
- [ ] Positive feedback from pet owners after referrals
- [ ] Other

Other

C4.

How many referrals do you make on average per week? ***(Numerical input)***

Please estimate approximately how many referrals you make per week.

C5. **Which referral method do you use most frequently?** ***(Multiple selections possible)***

- By telephone ☐
- In writing (letter) ☐
- In writing (fax) ☐
- Electronically (e-mail) ☐
- Electronically (web-based transfer platform) ☐
- Electronic (mobile app) ☐
- Other ☐

Other

[ ]

C6. **How much time does it take to process a referral?** ***(Numerical input in minutes)***

[ ]

C7.

How do you feel about the time required for a referral? ***(Single selection)***

- Too long ☐
- Appropriate ☐
- Other ☐

Other

[ ]

C8. **What information do your referrals usually contain?** ***(Multiple selections possible)***

- Name and contact details of the patient owner ☐
- Medical history ☐
- Diagnostic/treatment already performed ☐
- Medication ☐

LimeSurvey

Specific clinical questions to be answered ☐

Other ☐

Other

C9. **How do you rate the cooperation with referral practices?** ***(1-10, extremely poor to extremely good)***

1 – Extremely poor	2 – Very poor	3 - Poor	4 - Rather poor	5 - Fair	6 - Acceptable	7 – Rather good	8 - Good	9 – Very good	10 - Excellent
☐	☐	☐	☐	☐	☐	☐	☐	☐	☐

C10. **How easy or difficult is it to find a suitable referral practice?** ***(1-10, extremely difficult to extremely easy)***

1- Extremely difficult	2 - Very difficult	3 – Difficult	4 - Rather difficult	5 – Fair	6 - Accept-able	7 - Rather easy	8 - Easy	9 – Very easy	10 – Extremely easy
☐	☐	☐	☐	☐	☐	☐	☐	☐	☐

C11. **Which three aspects are particularly important to you in your collaboration?** ***(Multiple selections possible)***

Which of these aspects would you like to see in the specialist centre/clinic?

Good communication with the referral practice ☐

Timely discharge referral letters (to the first opinion practice) ☐

Appropriate treatment of the patient owner ☐

Professional skills ☐

Proximity to your own practice ☐

Transparent reporting ☐

Other ☐

Other

LimeSurvey

C12. **Which type of specialist do you prefer to refer patients to?** ***(Single selection)***

- Diplomate (European Board of Specialisation, EBVS) ☐
- Diplomate (American Board of Specialisation, ABVS) ☐
- “Fachtierarzt” (German specialist title) ☐
- All of the above ☐
- Doesn´t matter to me ☐
- Other ☐

Other

C13.

Please state the 3 most important reasons why you refer patients: ***(multiple selections possible)***

- Specialised diagnostics (except imaging procedures) ☐
- Specialised imaging procedures ☐
- Specialised medical therapy ☐
- Performance of a specialised surgical procedure ☐
- Lack of technical equipment ☐
- Time constraints ☐
- Wish of the patient's owner ☐
- Specialised knowledge, specific skills available ☐
- Confirmation of the diagnosis ☐
- Other ☐

Other

LimeSurvey

C14.

In your opinion, who is most competent in the field of clinical practice? ***(Single selection)***

- Diplomate (EBVS) ☐
- Diplomate (ABVS) ☐
- "Fachtierarzt" (German specialist title) ☐
- All of the above are equally competent in my opinion ☐
- Other ☐

Other

[ ]

C15.

In your opinion, who is most competent in veterinary medicine teaching? ***(Single selection)***

- Diplomate (EBVS) ☐
- Diplomate (ABVS) ☐
- "Fachtierarzt" (German specialist title) ☐
- None of the above ☐
- Other ☐

Other

[ ]

C16.

How many of the patients you refer are referred back by the referral practice/clinic for further treatment? ***(Numerical input in %)***

[| | | | | | | | |]

LimeSurvey

C17.

What information is particularly important to you when receiving a discharge letter? ***(1-10, extremely unimportant to essential)***

	1- Extremely unimportant	2 - Very unimportant	3 - Unimportant	4 - Rather unimportant	5 – Moderately unimportant	6 – Moderately important	7 – Rather important	8 - Important	9 – Very important	10 - Essential
Answers to specific questions	☐	☐	☐	☐	☐	☐	☐	☐	☐	☐
Specialist assessment of the patient	☐	☐	☐	☐	☐	☐	☐	☐	☐	☐
Results of tests and procedures	☐	☐	☐	☐	☐	☐	☐	☐	☐	☐
Clear communication about proposed or initiated therapy	☐	☐	☐	☐	☐	☐	☐	☐	☐	☐

C18. Which specialty area is most frequently involved in your referrals? ***(Single selection)***

- Dermatology ☐
- Gastroenterology ☐
- Cardiology ☐
- Neurology ☐
- Oncology ☐
- Surgery ☐
- Orthopedics ☐
- Radiology/Imaging ☐
- Ophthalmology ☐
- Dentistry ☐
- Reproductive medicine ☐
- Emergency and intensive care medicine ☐
- Other ☐

Other

C19 **Which cases do you refer most frequently?** ***(Free text)***

C20. **On a scale of 1 to 10, how likely are you to recommend the specialist to a colleague?** ***(1-10, extremely unlikely to extremely likely)***

1 – Extremely unlikely	2 – Very unlikely	3 – Unlikely	4 – Rather unlikely	5 – Moderately unlikely	6 – Moderately likely	7 – Rather likely	8 – Likely	9 – Very likely	10 - Extremely likely
☐	☐	☐	☐	☐	☐	☐	☐	☐	☐

C21.

What else is important to you in the referral process? ***(Free text)***

Part D: Question group 3 – Referral practice/clinic

D1.

How many referrals do you receive on average per week?
(Numerical input)

Please estimate how many referrals you receive from veterinary practices per week.

D2.

What information do referrals to you usually contain?
(Multiple selections possible)

- Name and contact details of the patient's owner ☐
- Medical history ☐
- Previous diagnostic/treatment ☐
- Medication ☐
- Specific clinical questions to be answered ☐

Other

☐

D3 **How important do you consider the following information to be when making a referral?** ***(1-10, extremely unimportant to essential)***

	1 – Extremely unimportant	2 - Very unimportant	3 – Un-important	4 – Rather unimportant	5 – Moderately unimportant	6 – Moderately important	7 – Rather important	8 - Important	9 – Very important	10 - Essential
Name and contact details of the patient owner	☐	☐	☐	☐	☐	☐	☐	☐	☐	☐
Medical history	☐	☐	☐	☐	☐	☐	☐	☐	☐	☐
Medication	☐	☐	☐	☐	☐	☐	☐	☐	☐	☐
Specific clinical questions to be answered	☐	☐	☐	☐	☐	☐	☐	☐	☐	☐

D4. **How do you rate the cooperation with the first opinion practices?** ***(1-10, extremely poor to extremely good)***

1 – Extremely poor	2 – Very poor	3 - Poor	4 - Rather poor	5 – Fair	6 - Acceptable	7 – Rather good	8 - Good	9 – Very good	10 - Excellent
☐	☐	☐	☐	☐	☐	☐	☐	☐	☐

D5.

Based on your practical experience, what percentage of referred pet owners do not want their regular vet to receive information about the appointment? ***(Numerical input in %)***

☐

D6. **In your opinion, how could cooperation be improved?** ***(Free text)***

☐

D7. **Which three aspects are particularly important to you in this collaboration?** ***(Multiple selection)***

Good communication with the first opinion practice ☐

Timely discharge referral letters (to the first opinion practice) ☐

LimeSurvey

- Appropriate treatment of pet owners ☐
- Professional skills ☐
- Proximity to your own practice ☐
- Transparent reporting ☐
- Trust ☐

D8. **On a scale of 1 to 10, how likely are you to recommend the GP to a colleague?** ***(1-10, extremely unlikely to extremely likely)***

1 – Extremely unlikely	2 – Very unlikely	3 – Unlikely	4 – Rather unlikely	5 – Moderately unlikely	6 – Moderately likely	7 – Rather likely	8 – Likely	9 – Very likely	10 - Extremely likely
☐	☐	☐	☐	☐	☐	☐	☐	☐	☐

D9.

Why are patients referred to you? ***(Multiple selections possible)***

- Specialised diagnostics (except imaging procedures) ☐
- Specialised imaging procedures ☐
- Specialised medical therapy required ☐
- Performance of specialised surgical procedures ☐
- Lack of technical equipment ☐
- Time constraints ☐
- Wish of the patient owner ☐
- Specialised expertise, specific skills available ☐
- Confirmation of the diagnosis ☐
- Other ☐

Other

☐

D10

How many of the patients you treat are discharged back to their GP for further treatment? ***(Numerical input in %)***

☐

D11.

Based on your practical experience, what percentage of referred pet owners do not wish to be discharged to their GP? ***(Numerical input in %)***

Part E: Question group 4 – General assessment of the referral process

E1.

What problems occur most frequently in the referral process?
(Multiple selections possible)

- Insufficient communication ☐
- Delayed discharge letter ☐
- Missing information ☐
- Patient owner dissatisfaction ☐
- Other ☐

Other

E2.

What suggestions do you have for improving the referral process? ***(Free text)***

You have reached the end. Thank you for participating in the survey! Your participation will help improve work processes in the veterinary field.

Corinna Gast (Doctoral student) Prof. Holger Volk Dr Claudia Busse Dr Christin Kleinsorgen
